# The Heterochronic Genes *lin-28a* and *lin-28b* Play an Essential and Evolutionarily Conserved Role in Early Zebrafish Development

**DOI:** 10.1371/journal.pone.0088086

**Published:** 2014-02-06

**Authors:** Yasuo Ouchi, Jyunya Yamamoto, Takashi Iwamoto

**Affiliations:** 1 The Center for Education in Laboratory Animal Research, Chubu University, Kasugai, Aichi, Japan; 2 Department of Biomedical Sciences, College of Life and Health Sciences, Chubu University, Kasugai, Aichi, Japan; Laboratoire de Biologie du Développement de Villefranche-sur-Mer, France

## Abstract

The *Caenorhabditis elegans* heterochronic gene pathway, which consists of a set of regulatory genes, plays an important regulatory role in the timing of stage-specific cell lineage development in nematodes. Research into the heterochronic gene pathway gave rise to landmark microRNA (miRNA) studies and showed that these genes are important in stem cell and cancer biology; however, their functions in vertebrate development are largely unknown. To elucidate the function of the heterochronic gene pathway during vertebrate development, we cloned the zebrafish homologs of the *C. elegans let-7* miRNA-binding protein, Lin-28, and analyzed their function in zebrafish development. The zebrafish genome contains two *Lin28*-related genes, *lin-28a* and *lin-28b*. Similar to mammalian Lin28 proteins, both zebrafish Lin-28a and Lin-28b have a conserved cold-shock domain and a pair of CCHC zinc finger domains, and are ubiquitously expressed during early embryonic development. In a reciprocal fashion, the expression of downstream heterochronic genes, *let-7* and *lin-4/miR-125* miRNA, occurred subsequent to *lin-28* expression. The knockdown of Lin-28a or Lin-28b function by morpholino microinjection into embryos resulted in severe cell proliferation defects during early morphogenesis. We found that the expression of *let-7* miRNA was upregulated and its downstream target gene, *lin-41*, was downregulated in these embryos. Interestingly, the expression of *miR-430*, a key regulator of maternal mRNA decay, was downregulated in *lin-28a* and *lin-28b* morphant embryos, suggesting a role for Lin-28 in the maternal-to-zygotic transition in zebrafish. Taken together, our results suggest an evolutionarily conserved and pivotal role of the heterochronic gene pathway in early vertebrate embryogenesis.

## Introduction

The spatial and temporal coordination of gene expression during development is essential for the correct morphogenesis of animals. Genetic studies of *Caenorhabditis elegans (C. elegans)* have provided significant insight into the evolutionarily conserved networks of gene regulation that underlie development [1]. The *C. elegans* heterochronic gene pathway, the study of which led to the landmark discovery of the first microRNA (miRNA)-mediated gene regulatory network during development, was also discovered through conventional forward genetic screens in *C. elegans* for heterochronic (developmental timing) mutants [2]–[6].

miRNAs are a class of post-transcriptional regulatory noncoding small RNAs (18–25 nucleotides in length) that bind specific target sequences, usually located in the 3′-untranslated region (UTR) of regulated mRNAs, and block their translation and/or initiate their degradation [7], [8]. In vertebrate miRNA biogenesis, the primary transcripts of miRNA genes (pri-miRNAs) are cleaved into short hairpin intermediates (pre-miRNAs) by the nuclear RNase III Drosha and the Dgcr8 complex, and are further processed to mature miRNAs by the cytosolic RNase III-related enzyme Dicer. Then, the mature miRNA molecule binds to an RNA-induced silencing complex, which regulates protein expression [9].

The functional significance of miRNAs in vertebrate development has been clearly demonstrated using Dicer mutants [10], [11]. In *Danio rerio* (zebrafish), embryos deficient in maternal and zygotic Dicer activity (MZ *dicer* mutants) cannot generate mature miRNAs and display severe morphogenetic defects [12]. For example, MZ *dicer* mutants develop more slowly than wild-type embryos and have reduced extension of the body axis, resulting in shortening of the embryo and morphological malformation of the heart and brain [12]. Interestingly, the injection of *miR-430*, which has a crucial function in deadenylation and the clearance of maternal mRNAs, rescues these brain defects, suggesting a critical role for *miR-430* in early zebrafish development [12].

In the *C. elegans* heterochronic pathway, *let-7* and *lin-4/miR-125* miRNA play an essential regulatory role in the timing of stage-specific cell lineage development in nematodes, in part by directly regulating their target genes [5], [6], [13], [14]. For example, *lin-4/miR-125* miRNA promotes the transition between the first and second larval stages (L1 and L2, respectively) by binding to complementary sites in the 3′UTR of the upstream heterochronic genes *lin-14* and *lin-28* [3], [6], [15]–[17]. Since LIN-28 prevents the premature accumulation of *let-7* miRNA in L2, *let-7* miRNA expression occurs during the third larval stage (L3) and controls the fourth larval stage (L4)-to-adult transition by repressing multiple target genes, including the TRIM protein Lin-41 [6], [18], [19]. Interestingly, recent works have shown that several members of the heterochronic pathway are highly conserved by sequence and play a critical role in humans and mice [20]–[22]. However, it is unknown whether these heterochronic homologs function to regulate the timing of specific developmental events in vertebrates.

In this study, to elucidate the function of the heterochronic pathway during vertebrate development, we used zebrafish as an animal model to investigate the role of Lin-28 during development. We cloned two zebrafish homologs of *lin-28*, *lin-28a* and *lin-28b*, and analyzed their functions in development. Zebrafish *lin-28a* and *lin-28b* were ubiquitously expressed in early embryonic development, and their expression decreased at 12 h post-fertilization (hpf) and 24 hpf, respectively. On the other hand, the expression of *let-7* and *lin-4/miR-125b* miRNA, a downstream heterochronic gene of *lin-28*, was expressed subsequent to *lin-28b* expression. The knockdown of Lin-28a or Lin-28b function by morpholino (MO) microinjection resulted in severe cell proliferation defects during early morphogenesis, and the expression of both *let-7* and *lin-41* was modulated. Interestingly, a microarray-based analysis of miRNAs showed that *miR-430* expression was inhibited in *lin-28a* and *lin-28b* morphant embryos, suggesting that the clearance of maternal mRNAs was affected in these morphants. These results suggest an evolutionarily pivotal role of Lin-28 for the heterochronic pathway in early vertebrate embryogenesis, and provide novel insight into Lin28 function.

## Materials and Methods

### Fish Handling and Care

Wild type zebrafish strains AB were maintained according to The zebrafish book: A Guide for the Laboratory Use of Zebrafish. All animals were handled in strict accordance with good animal practice as defined by national and local animal welfare bodies, and all efforts were made to minimize suffering. All animal work was approved by the the Committee on the Ethics of Animal Experiments and the Institutional Animal Care and Use of Chubu University; serial permit numbers were not necessarily assigned to fish experiments in Chubu University.

### Genomic Analysis

We used the VISTA Web server to align genomic sequences (http://www.gsd.lbl.gov/VISTA/). All the genomic sequences were found by Ensembl and ZFIN database search and the coordinates are as follows: Homo sapiens (human) *LIN28A*, chromosome 1, 26,737,269–26,756,213 bp, human *LIN28B*, chromosome 6, 105404923–105531207 bp, Gallus gallus (Chicken) *LIN28A*, chromosome 23, 148,871–160,164 bp, Gallus gallus *LIN28B*, chromosome 3, 71550790–71626325 bp, Mus muculus (Mouse) *Lin28A*, chromosome 4, 134,003,330–134,018,841 bp, *Mouse Lin28B*, chromosome 10, 45,376,620–45,470,201 bp, Danio rerio (zebrafish) *lin-28a*, chromosome 19, 14873056–14901221 bp, Danio rerio *lin-28b*, chromosome 20, 47,677,565–47,707,773 bp.

Transposable elements were masked prior to submission using the RepeatMasker program, and the annotated mouse sequence was used as the base reference track. Pair-wise sequence comparisons were calculated with a threshold of 70% identity in a 10-bp window, with a 0% minimum identity shown.

### Cloning and RT-PCR

RT-PCR was performed as previously described [27]. Briefly, total RNA was purified from zebrafish embryos (TRIzol reagent; Invitrogen-Gibco, Carlsbad, CA), and cDNA was synthesized (Superscript II; Invitrogen-Gibco). The primer sets were tested over a range of thermal cycles using ExTaq (Takara, Shiga, Japan), and the semiquantitative cycle number was determined for each primer set. Bands were visualized with ethidium bromide. The primer sequences used for the PCR were shown 5′ to 3′:

Lin41Fw: 5′-ATGGCTTCGTTTCCAGACTC-3′, Lin41Rv: 5′-TCATGTCCCTCACATTCTAC-3′, Lin28AFw: 5′-TAAAGATGCCCCCGGCAAAT-3′, Lin28ARv: 5′-CTCTGCTAATCAGTGCTCTC-3′, Lin28ARv2: 5′-GAGCCGTGAAAAGAGCCTGA-3′(For knockdown analysis), Lin28BFw: 5′-TTCGCTGGAACTTTGGAACG-3′, Lin28BRv: 5′-TTCAGTCCCGGCTCTTTCTC-3′, Lin28BRv2: 5′-TGCGGACATTGAACCACTTG-3′ (For knockdown analysis), Sox2Fw: 5′-AAGGAACACCCGGATTACAA-3′, Sox2Rv: 5′-TCATGTCAGCCTTTGCAGAA-3′, Otx2Fw: 5′-ATGATGTCGTATCTCAAGCA-3′, Otx2Rv: 5′-CTTGGTCCTTATAATCCAAG-3′, gapdhFw: 5′-CTGCCAAGGCTGTGGGCAAG-3′, gapdhRv: 5′-TTACTCCTTGGAGGCCATGT -3′,

The amplified PCR products were subcloned into pCS2+ or pGEM-T easy (Promega) vector and confirmed by sequencing analysis. Amino acid sequence comparisons and phylogenic tree analysis were carried out with APE and Jalview software. The sequence was submitted to GenBank (Accession number; AB828400).

### Whole Mount *in situ* Hybridization

Whole-mount *in situ* hybridization was done as previously described [28]. Digoxigenin (DIG)-labeled sense and antisense RNA probes were made by *in vitro* transcription with T7 or SP6 RNA polymerase (Roche Diagnostics GmbH, Mannheim, Germany), using templates generated by PCR. Embryos were fixed in 4% paraformaldehyde (PFA) and stored in methanol. Rehydrated embryos were sequentially treated with Proteinase K and then acetylated in acetic anhydrate solution. The pretreated samples were next hybridized at 60°C with DIG-labeled RNA probe. The probe was detected with an anti-DIG antibody-conjugated to alkaline phosphatase (Roche Diagnostics GmbH, Mannheim, Germany) and visualized by using a BCIP/NBT solution kit (Nacalai Tesque Inc.).

### Reverse Transcription and Real-time PCR Quantification of miRNA

For miRNA quantification, cDNA was synthesized from total RNA using gene-specific primers according to the TaqMan MicroRNA Assay protocol as per the manufacturer’s protocol (Applied Biosystems). Quantitative PCR of miRNA was performed using an Applied Biosystems 7300 Sequence Detection system. The 10 µl PCR reaction contained 0.67 µl RT product, 1 × TaqMan Universal PCR master mix, and 1 µl of the primer and probe mix, according to the TaqMan MicroRNA Assay protocol (Applied Biosystems). The threshold cycle data were determined using default threshold settings. The threshold cycle was defined as the fractional cycle number at which the fluorescence exceeded the fixed threshold. Gapdh or *miR-26a* were used as internal control.

### Microinjection of Morpholinos or rescueRNA into Zebrafish Embryos

Antisense oligonucleotide Morpholinos (MO) were designed and obtained from Gene Tools, LLC. The *lin-28a* and *lin-28b* splicing MOs were designed splicing donor region of intron between exon 1 and exon 2. The sequences of the MO were shown 5′ to 3′: *lin-28a* MO: 5′-GAAGTTCTCACCTGTGTGATTGAGA-3′, *lin-28b* MO: 5′-AAAGCATCGTAACCTTCGGCCATGT-3′, Control MO: 5′-CCTCTTACCTCAGTTACAATTTATA-3′.

For RNA synthesis, capped sense RNAs were synthesized by using an mMessage mMachine in vitro transcription kit (Ambion, Austin, TX) according to the manufacturer's instructions. The synthesized RNAs were diluted to an appropriate concentration with RNase-free water.

In all experiments, MOs and mRNAs were resuspended in H_2_O with Phenol red (0.05%). Fertilized eggs from wild-type zebrafish at the one- to two-cell stage were injected with approximately 1–3 nl of the solution (10 µM) by using a microinjector (IM-300; Narishige) as described elsewhere [29].

### BrdU Labeling and Immunodetection

For BrdU incorporation, 4 hours postfertilization (hpf) embryos were treated with 10 mM BrdU in egg water for 60 minutes and washed two times with egg water, followed by 4% paraformaldehyde fixation overnight at 4°C. After gradual rehydration, embryos were incubated in 2N HCl for 1 hour at 37°C. After rinsing with PBS, they were permeabilized with 0.1% Triton X/PBS, washed with PBS, blocked with 2% BSA/PBS for at least 1 hour at room temperature, and incubated with rat anti-BrdU antibody (Roche) in 2% BSA/PBS overnight at 4°C. After washing three times with PBS for 5 minutes each, anti-BrdU antibody was visualized using secondary antibodies conjugated with Alexa Fluor 594 (Molecular Probes). Nuclei were counterstained with DAPI. Embryos were mounted in 1% agarose and confocal fluorescence images were obtained using a confocal microscope (FV-1000; Olympus).

### Microarray Analysis

RNA was isolated using Trizol from control MO, *lin-28a* MO and *lin-28b* MO injected embryos at 5hpf. For miRNA expression analysis, 250 ng of total RNA was subjected to microarray analysis using a miRCURY LNA microRNA Array (Exiqon). Labeling, hybridization, washing, and scanning of the microarray were performed by Cosmo Bio Co., Ltd. (Tokyo, Japan), following the manufacturer’s instructions. Data analyses were performed using the Mev software (MultiExperiment Viewer). The experimental data used in this manuscript are freely available in the NCBI gene expression omnibus (GEO) repository with the accession number GSE52492.

### Statistical Analysis

Data were analyzed by the two-tailed Student’s *t*-test. Values were expressed as mean ± SEM. Changes were deemed significant if the *p*-value was <0.05. Statistical significance is indicated as follows: * *p*<0.05, ** *p*<0.01, *** *p*<0.001, N.S., not significant.

## Results

### Cloning of *Lin28* Homologs in Zebrafish

**Figure 1:**
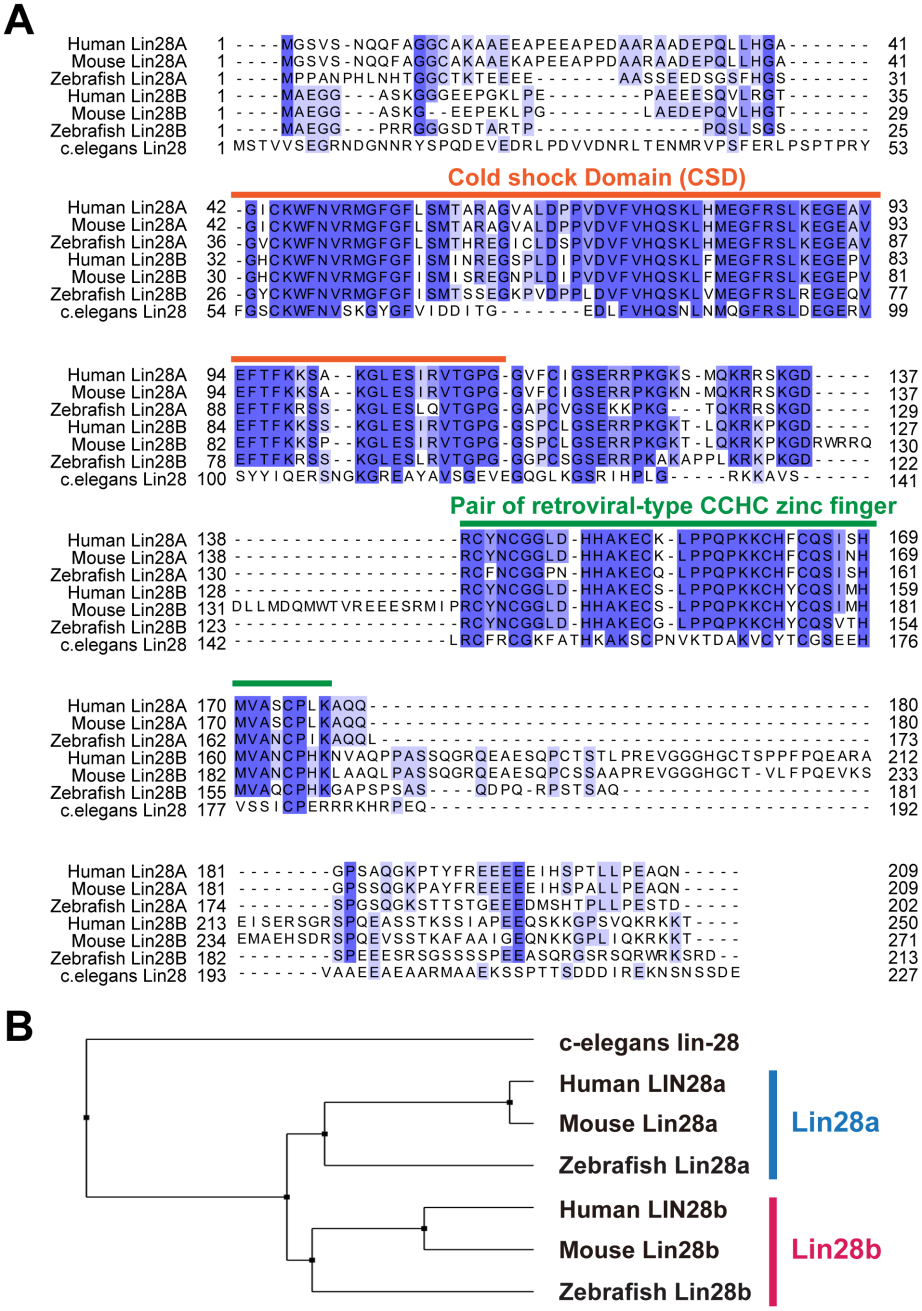
Cloning and characterization of zebrafish Lin-28a and Lin-28b. A. Deduced amino acid sequences of zebrafish Lin-28a and Lin-28b aligned against human, mouse and c.elegans Lin28 homologs. Blue boxes represent identical and similar amino acids. The characteristic Lin28 structure including cold-shock domain and two CCHC-Zn finger domains was highly conserved in zebrafish Lin-28a and Lin-28b. B. The phylogeny of the Lin28 family based on the alignment of full length of amino acid sequence. The branch length (X axis) in the rectangular cladogram represent the distances among those sequences calculated using BLOSUM62 substitution matrix.

To analyze the developmental function of Lin28 using zebrafish as a model system, we first searched the zebrafish Ensembl Genome Server (www.ensembl.org/Danio_rerio/) for sequences homologous to human *Lin28a* and *Lin28b*, which were identified in mammalian genomes as two homologs of the *C. elegans* heterochronic gene *lin-28*, *Lin28a* and *Lin28b* [30], [31]. However, since the zebrafish *Lin28* homolog was not fully assigned in the database, we only found one *Lin28* homolog at Chromosome (Chr.)19 and a partially expressed sequence tag at Chr. 20. To clone and examine these genes, we first used VISTA genome alignment tools to align and compare the sequences of their corresponding genomic loci with those from other species, including human, rat, mouse, and chicken. As a result, we identified a highly conserved candidate genomic region of zebrafish *lin-28a* and *lin-28b* at Chr. 19 and Chr. 20, respectively (Figure S1A and B). Polymerase chain reaction (PCR) primers were designed using sequence information from the surrounding conserved exons and amplified PCR products were sequenced to obtain full-length zebrafish *lin-28a* and *lin-28b*. The deduced amino acid sequences of *lin-28a* and *lin-28b* encoded 202 and 213 amino acids, respectively. Two domains containing RNA-binding motifs, an N-terminal cold-shock domain and a pair of retroviral-type CCHC zinc fingers near the C-terminus, presumably play an important role in *let-7* miRNA binding [32]. Since the cold-shock domain and retroviral-type CCHC finger regions of zebrafish Lin28A and Lin28B had 79–90% homology at the amino acid level with corresponding regions in mouse and human LIN28A and LIN28B (Figure 1A), the function of Lin-28a and Lin-28b may be conserved across animal phylogeny. Furthermore, a phylogenetic analysis suggested that these two Lin-28 proteins belong to the LIN28A and LIN28B groups (Figure 1B), as expected.

### Expression Pattern of Heterochronic Genes during Zebrafish Development

**Figure 2:**
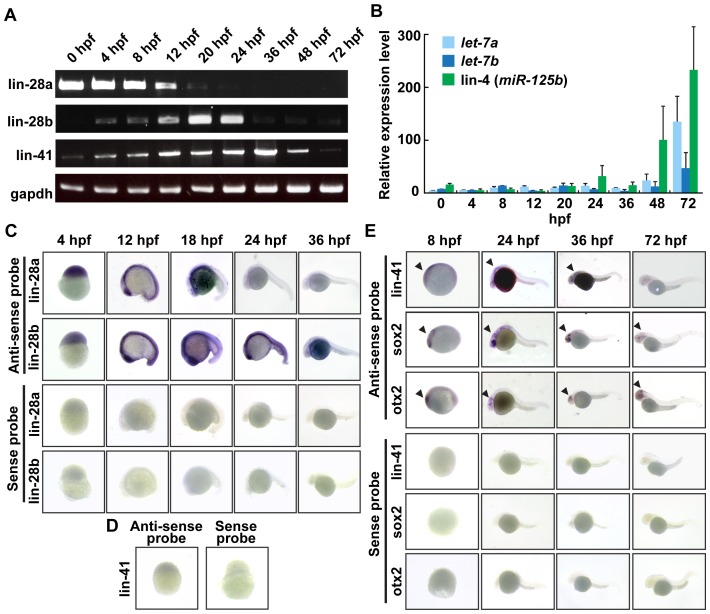
Expression patterns of zebrafish heterochronic genes during embryonic development. A. Semiquantitative RT-PCR analysis of *lin-28a, lin-28b and lin-41* gene mRNA expression. Total RNA was isolated from wild-type embryos at the indicated developmental stages and analyzed by using specific primers for *lin-28a*, *lin-28b* and *lin-41*. *gapdh* was used as a control. B. Real-time PCR analysis of *let-7a*, *let-7b* and *lin-4/miR-125b* miRNA expression. Total RNA was isolated from wild-type embryos at the indicated developmental stages. Expression levels of *let-7a*, *let-7b* and *lin-4/miR-125b* was analyzed using TaqMan miRNA assay. C. Developmental expression analysis of zebrafish *lin-28a* and *lin-28b* mRNAs by whole mount in situ hybridization. Zebrafish embryos at various stages were hybridized with antisense probes. D, E. Developmental expression analysis of zebrafish *lin-41* mRNAs by whole mount in situ hybridization. *sox2* and *otx2* probes were used as a CNS maker. Arrowhead indicated expression in the CNS.

To characterize the expression pattern of zebrafish homologs of heterochronic genes (including *lin-28*, *let-7*, *lin-41/TRIM71*, and *lin-4/miR-125b*) in zebrafish development, reverse transcription-PCR (RT-PCR), TaqMan quantitative RT-PCR (qRT-PCR), and whole-mount *in situ* hybridization were performed on embryos at various developmental stages. First, RT-PCR analyses of *lin-28a*, *lin-28b*, and *lin-41* mRNA expression during development showed strong *lin-28a* expression from the one-cell stage until 30% epiboly and then gradually decreased at 12 hpf, indicating that *lin-28a* is maternally expressed (Figure 2A). In contrast, *lin-28b* was expressed subsequent to *lin-28a* expression, and gradually increased after fertilization with a peak at 20 hpf and then decreased (Figure 2A). Faint *lin-41* expression was first detected at 0 hpf; expression gradually increased to its peak at 36 hpf (Figure 2A). After 48 hpf, *lin-41* expression began to decrease and was undetectable at 72 hpf (Figure 2A). Interestingly, consistent with decreased *lin-28a* and *lin-28b* expression, *let-7a, let-7b*, and *lin-4/miR125b* miRNA expression was dramatically increased at 72 hpf (Figure 2B).

We next examined the spatial distribution of *lin-28a*, *lin-28b*, and *lin-41* mRNA in developing embryos by whole-mount *in situ* hybridization using DIG-labeled RNA as a probe. A hybridization signal for both *lin-28a* and *lin-28b* was observed in the entire embryonic region of eggs at 4 hpf, whereas a very faint or no *lin-41* signal was detected (Figure 2C and Figure 2D). Later, while a *lin-28b* mRNA signal was observed throughout the entire body until 24 hpf, *lin-28a* expression disappeared from the caudal half of the embryos and was undetectable after 24 hpf (Figure 2C). At 36 hpf, we did not observe significant expression of *lin-28a* or *lin-28b* mRNA, suggesting that *lin-28a* and *lin-28b* play a role in early development. On the other hand, a relatively strong *lin-41* mRNA signal began to appear in the anterior neural plate at 8 hpf, where the earliest neural molecular markers, *sox2* and *otx2*, were expressed (Figure 2E). At 24–36 hpf, *lin-41* was restricted to the entire anterior half of the embryo, where the *sox2* and *otx2* were also expressed, and become undetectable by 72 hpf (Figure 2E). These expression patterns suggest that an evolutionarily conserved heterochronic gene regulatory hierarchy is used even in vertebrate development.

### *Lin-28a* and *Lin-28b* are Required for Proper Gastrulation in Zebrafish Embryos

**Figure 3:**
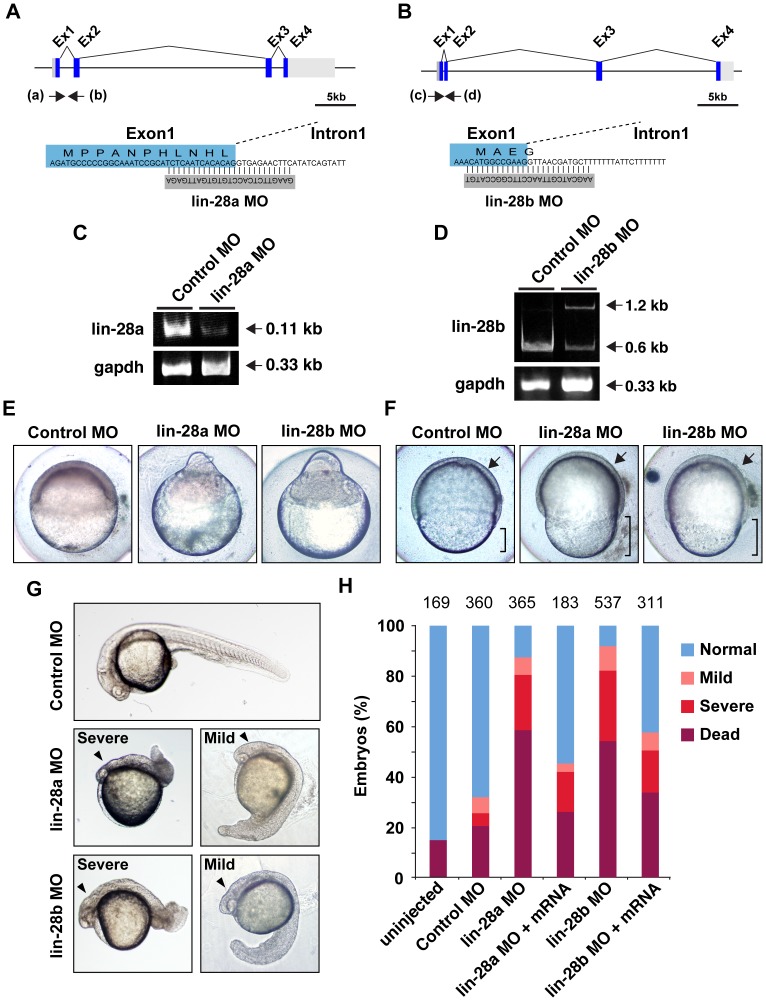
Morpholino-mediated knockdown of *lin-28a* and *lin-28b* affects early development. A. B. Schematic representation shows the genomic organization of the *lin-28a* and *lin-28b* genes in zebrafish. Regions targeted by splice-blocking morpholinos are shown. Arrows depict the location of the two primer sets to amplify exon and intron sequences, which were utilized in the RT-PCR analysis. C. D. The efficacy of MO was validated by RT-PCR using primers as indicated in A, B. Total RNA was isolated from *lin-28a*, *lin-28b* or control MO-injected embryos at 5 hpf and analyzed by using specific primers for *lin-28a* and *lin-28b*. Upper panel shows a reduced expression of *lin-28a* and *lin-28b mRNA*, and an increase of an aberrantly spliced *lin-28b* transcripts corresponding to the presence of intron 1. Lower panel shows RT-PCR products with *gapdh* primers used as internal control. E. Representative images of severe phenotype of *lin-28a* and *lin-28b* morphants at 5 hpf. F. Vegetal view of control, *lin-28a* and *lin-28b* morphants at 8 hpf. Arrow shows the similar extent of prechordal plate extension in control, *lin-28a* and *lin-28b* morphant embryos; bracket shows a reduced extent in epiboly in *lin-28a* and *lin-28b* morphant embryos compared with controls. G. Representatives of mild and severe phenotypes in *lin-28a* and *lin-28b* morphants at 30 hpf. Both *lin-28a* and *lin-28b* morphants develop morphological phenotypes displaying shorten body axis, small anterior structures and aberrant tail morphology ranging from mild (Right) to severe (Left) compare to control MO injected embryos (Top). Arrowheads indicate the smaller head. H. Quantification of the efficiency of rescue from gastrulation defects following co-injection of *lin-28a* MO, *lin-28b* MO and mRNAs. More than half of the *lin-28a* MO or *lin-28b* MO injected embryos had died by 30 hpf. The frequencies of the phenotypes were similar between *lin-28a* and *lin-28b* morphants. The frequency of the dead, mild and severe phenotypes decreased with a rescue experiment in which 300 pg of *lin-28a* and *lin-28b* mRNAs were co-injected with the MO. (p<0.0001, *lin-28a* MO vs. *lin-28a* MO+*lin-28a* mRNA, *lin-28b* MO vs. *lin-28b* MO+*lin-28b* mRNA, Fisher’s exact test). The total number of embryos is noted above each bar.

To examine the role of *Lin-28a* and *Lin-28b* in the development of zebrafish embryos, we designed and injected MO oligonucleotides that blocked the normal splicing of *lin-28a* and *lin-28b* in fertilized eggs (Figure 3A and Figure 3B). To confirm the knockdown, we performed RT-PCR using *lin-28a*- and *lin-28b*-specific primer sets that amplified normal or abnormal transcripts with exon skipping (Figure 3A and Figure 3B). In 3 ng of *lin-28a* MO-injected embryos, although a band corresponding to aberrant splicing was not observed, the morphants displayed a significant decrease in lin-28A transcripts (Figure 3C). Similarly, in *lin-28b* MO-injected embryos, a band corresponding to normal splicing (0.6 kb) was reduced by *lin-28b* MO (Figure 3D). In addition, a band corresponding to aberrant splicing (1.2 kb) was observed in the *lin-28b* MO-injected sample (Figure 3D). These results suggest that the lin-28a and *lin-28b* MOs effectively knocked down *lin-28a* and *lin-28b* in zebrafish. As we expected, the *lin-28a* and *lin-28b* morphants displayed severe growth retardation and reduced epiboly as compared to the controls (Figure 3E and Figure 3F). Morphants of the most severe phenotype, failed to initiate blastoderm epiboly (Figure 3E). As a result, approximately 40% of the injected embryos died around 12 hpf. Later, both the remaining *lin-28a* and *lin-28b* morphants displayed reduced extension of the body axis, resulting in shortened embryos and a reduced head size. At 30 hpf, both the remaining *lin-28a* and *lin-28b* morphant embryos exhibited a severe phenotype in which the embryo had a very small head and tail (Figure 3G). The survival rates and phenotype percentages at 30 hpf are shown in Figure 3H. Both MO groups had a significant increase in death and abnormal morphology relative to control MOs (Figure 3H; p<0.0001, control MO vs. *lin-28a* MO, control MO vs. *lin-28b* MO, Fisher’s exact test); 58.4% of *lin-28a* morphants and 54.6% of *lin-28b* morphants exhibited the dead phenotype, while the percentage of dead in the control was 20.3%.

To ensure the specificity of the *lin-28a* and *lin-28b* MOs, we performed a rescue experiment. Co-injection of zebrafish *lin-28a* or *lin-28b* mRNA (300 pg) with the splice-blocking MO resulted in significant rescue of the gastrulation defect; the percentage of the “dead” phenotype was reduced from 58.4% in *lin-28a* MO-injected embryos to 25.7% and from 54.6% in *lin-28b* MO-injected embryos to 33.4% (Figure 3H). Moreover, the “severe” phenotype was also reduced in both mRNA co-injected embryos. This rescue experiment confirmed that both *lin-28a* and *lin-28b* are required for early morphogenesis in zebrafish.

### Both *Lin-28a* and *Lin-28b* Regulate Cell Proliferation and Neural Expansion during Early Development

**Figure 4:**
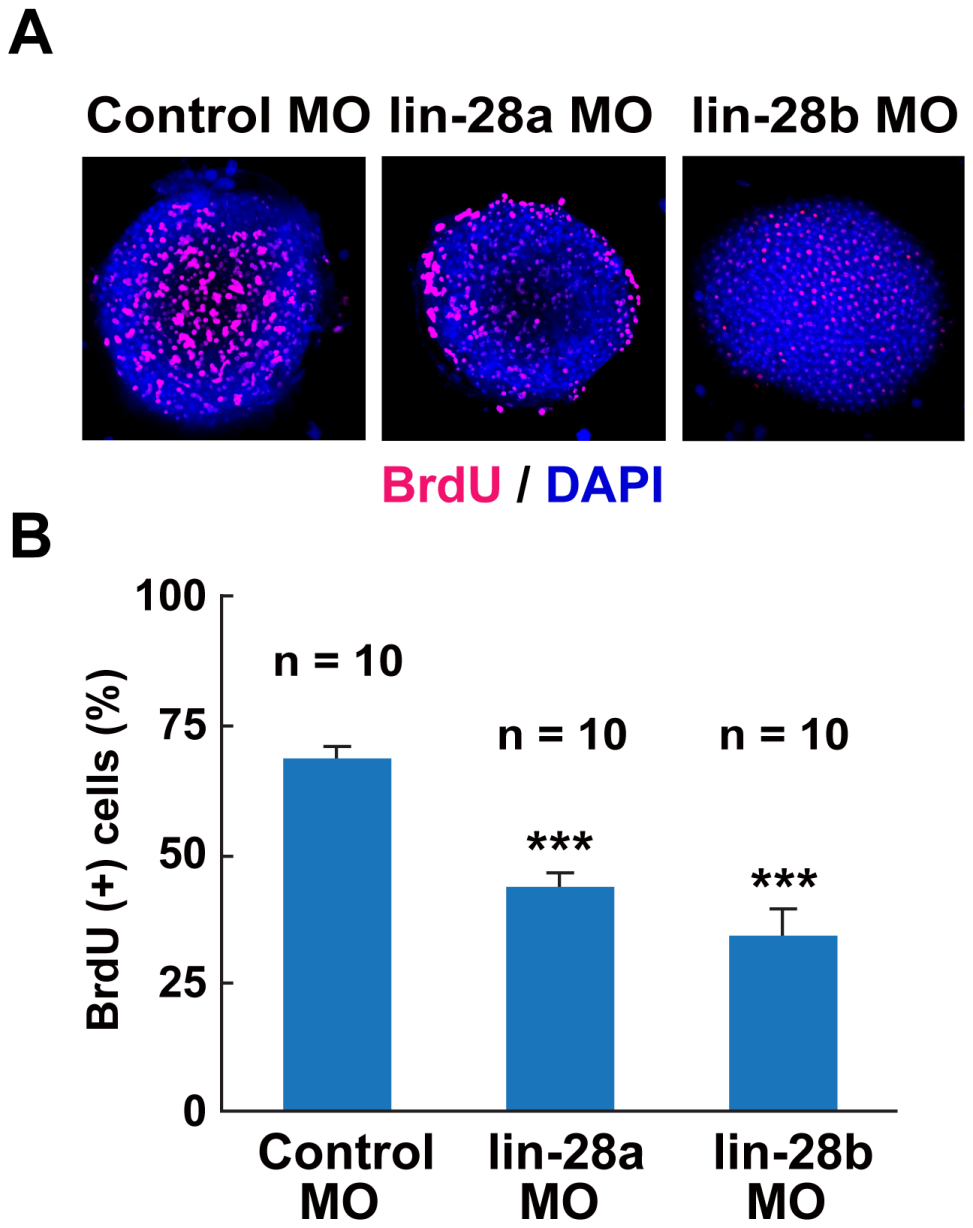
Effect of *lin-28a* and *lin-28b* knockdown on cell proliferation. A. Confocal images of BrdU (Red) incorporation in *lin-28a* and *lin-28b* morphant embryos at 5hpf. Both *lin-28a* and *lin-28b* MO-injected embryos were exposed to BrdU at 4 hpf for 1 hour. The nuclei were stained with DAPI (blue). B. Percentage of BrdU incorporated cells in *lin-28a* and *lin-28b* morphants embryos. The percentage of BrdU-incorporated cells was calculated by dividing the number of BrdU-positive cells by the total number of cells as determined by DAPI-stained cells. The percentage of BrdU-positive cells was significantly lower in both *lin-28a* MO- and *lin-28b* MO-injected embryos compared with control MO-injected embryos (***p<0.001, control MO vs. *lin-28a* MO, control MO vs. *lin-28b* MO, two-tailed test, n = 10, mean ± SEM).

*Lin28a* and *Lin28b* play a critical role in the proliferation of various mammalian cells, including ES cells, and in the formation of iPS cells [23]–[25]. Thus, to characterize the severe growth retardation phenotype observed in the early stage of *lin-28a* and *lin-28b* morphants, we investigated cellular proliferation in these morphants using BrdU. *Lin-28a* or *lin-28b* morphant embryos were treated with BrdU egg water at 4 hpf for 1 h and the percentage of BrdU-positive cells was examined by immunohistochemistry (Figure 4A). We observed a significant reduction in BrdU incorporation in both *lin-28a* and *lin-28b* morphants as compared with the controls (Figure 4A and B). These findings indicate that the severe morphological defects observed in the *lin-28a* and *lin-28b* morphants may have resulted from impaired cellular proliferation.

### *Lin-28a* and *Lin-28b* Regulate the Expression of Downstream Heterochronic Genes, as well as the *miR-430* miRNA family

**Figure 5:**
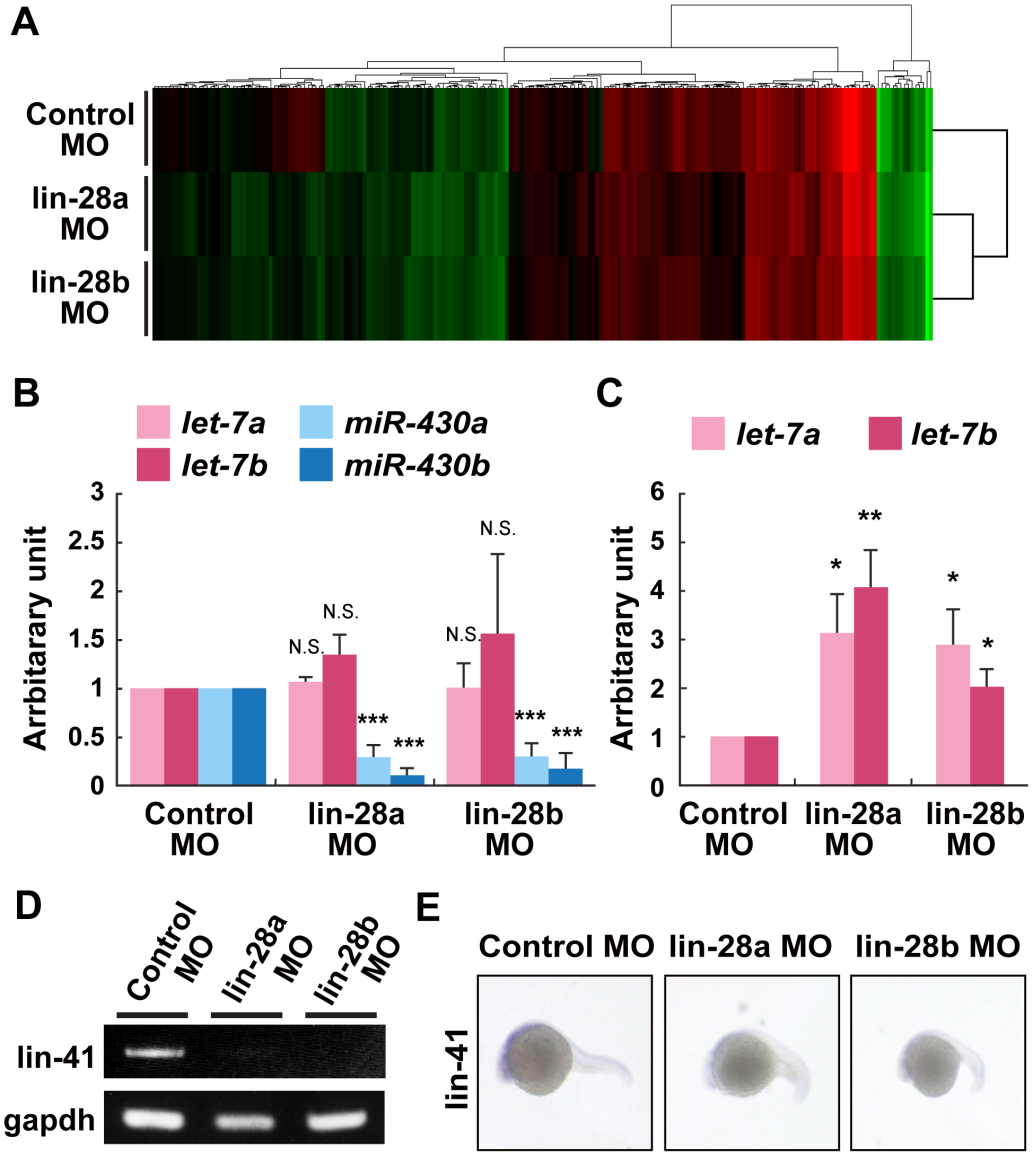
Expression of miRNAs and downstream heterochronic genes in *lin-28a* and *lin-28b* morphant embryos. A. miRNA expression profiling in *lin-28a* and *lin-28b* morphants embryos at 5 hpf. Hierarchically clustered heat-map representing differences in miRNA expression between control MO-injected embryos and *lin-28a* MO- or *lin-28b* MO-injected embryos. Relative levels of expression are colored from green (low) to red (high). B. Real-time PCR analysis of *let-7a, let-7b, miR-430a* and *miR-430b* expression in control MO-, *lin-28a* MO- and *lin-28b* MO-injected embryos. Expression levels of *let-7a, let-7b, miR-430a* and *miR-430b* were analyzed at 5 hpf using TaqMan miRNA assay. The expression level of *miR-430a* and *miR-430b* was downregulated in both *lin-28a* MO- and *lin-28b* MO-injected embryos compared with control MO-injected embryos (***p<0.001, control MO vs. *lin-28a* MO, control MO vs. *lin-28b* MO, two-tailed test, n = 3, mean ± SEM). C. Real-time PCR analysis of *let-7a* and *let-7b* expression in control MO-, lin-28a MO- and lin-28b MO-injected embryos. Expression levels of *let-7a* and *let-7b* were analyzed using TaqMan miRNA assay at 28 hpf. The expression level of *let-7a* and *let-7b* was increased in both *lin-28a* MO- and *lin-28b* MO-injected embryos embryos compared with control MO-injected embryos (*p<0.05, **p<0.01, control MO vs. *lin-28a* MO, control MO vs. *lin-28b* MO, two-tailed test, n = 4, mean ± SEM). D. Semiquantitative RT-PCR analysis of *lin-41* gene expression in control MO-, lin-28a MO- and lin-28b MO-injected embryos. Total RNA was isolated from *lin-28a* MO-, *lin-28b* MO- or control MO-injected embryos at 24 hpf and analyzed by using specific primers for *lin-41.* The expression of *lin-41* was decreased in both *lin-28a* MO- and *lin-28b* MO-injected embryos compared with control MO-injected embryos. E. Control, *lin-28a* and *lin-28b* MO-injected embryos labeled by *in situ* hybridization with *lin-41* probe. Expression of *lin-41* was repressed in CNS of both *lin-28a* and *lin-28b* morphant embryos at 24 hpf.

In the *C. elegans* heterochronic pathway, miRNAs, including *let-7* and *lin-4/miR-125*, play a critical role as downstream genes of *lin28*. To determine the downstream events following *lin-28* expression, we conducted an miRNA microarray analysis and examined the global changes in miRNA expression in *lin-28a* and *lin-28b* morphant embryos. Total RNA from control, lin-28a, and *lin-28b* MO-injected zebrafish embryos was isolated at 5 hpf and subjected to microarray analysis using an miRCURY LNA microRNA array for differential miRNA expression (Figure 5A). The heat map from the two-way hierarchical clustering analysis showed that most of the miRNAs on the microarray had a similar expression pattern among the samples, but that some of the miRNAs had substantial differences in their expression patterns to be clustered (Figure 5A). Additionally, the miRNA expression pattern in *lin-28a* MO-injected embryos clustered more closely with those in *lin-28b* MO-injected embryos than with those in control MO-injected embryos, suggesting that these two genes have comparable functions (Figure 5A).

To detect significant changes in miRNA expression, we chose miRNAs that had at least a 1.5-fold change with high Hy3 signal (sample) in expression in the *lin-28a* or *lin-28b* morphants as compared with control MO-injected embryos (Table 1). Using this criterion, several miRNAs, including *miR-203a*, were downregulated in both *lin-28a* and *lin-28b* morphant embryos. Interestingly, we found a marked decrease in the expression of members of the *miR-430* family, which was recently demonstrated to play a crucial role in deadenylation and clearance of maternal mRNAs during early zebrafish development (Table 1). To confirm this, we examined the expression level of these miRNAs by qRT-PCR (Figure 5B). Consistent with our microarray data, we observed a significant decrease in the expression of *miR-430a* and *miR-430b* (Figure 5B). Surprisingly, the expression level of *let-7* miRNA in *lin-28a* and *lin-28b* morphant embryos was very low, and we did not detect significant upregulation in these morphants, suggesting that the downregulation of Lin-28a or Lin-28b is itself insufficient to induce the expression of the *let-7* family, at least at 5 hpf (Figure 5B).

**Table 1:** List of differentially expressed probe sets (Fold change >1.5, %CV <50, High Hy3 signal >100).

Probe Name	Name	Hy3/Hy5			Fold change	
		Control MO	Lin28a MO	Lin28b MO	Lin28a MO/Control MO	Lin28b MO/Control MO
49301	dre-miR-203a	1.029	0.939	0.651	0.913	0.633
48937	dre-miR-430a	1.322	0.81	0.783	0.613	0.592
48938	dre-miR-430b	1.288	0.662	0.808	0.514	0.627
50268	dre-miR-430c	1.18	0.695	0.606	0.589	0.513
48939	dre-miR-430j	1.35	0.64	0.766	0.475	0.568

To further examine the expression level of factors downstream of *lin-28a* and *lin-28b*, we isolated total RNA from surviving control, *lin-28a*, and *lin-28b* MO-injected zebrafish embryos at 28 hpf, which is the stage just before the expression of *let-7a* and *let-7b* was observed, and performed qRT-PCR to determine the relative expression levels of these miRNAs. As expected, the miRNA expression of both *let-7a* and *let-7b* was increased two- to four-fold in *lin-28a* and *lin-28b* morphant embryos (Figure 5C).

Furthermore, since *lin-41* is a direct target of *let-7* in *C. elegans* and mice, we performed RT-PCR and *in situ* hybridization to investigate whether the expression of *lin-41* was downregulated in *lin-28a* or *lin-28b* morphant embryos. Consistent with a prominent increase in *let-7* miRNA, RT-PCR analysis of *lin-41* expression showed that *lin-41* mRNA was downregulated in both *lin-28a* and *lin-28b* morphants as compared to the control sample at 24 hpf (Figure 5D). *In situ* hybridization of *lin-41* mRNA confirmed that the hybridization signal in the anterior half of the embryos was barely detectable in the surviving *lin-28a* or *lin-28b* morphants at 24 hpf (Figure 5E). These results suggest the existence of an evolutionarily conserved heterochronic gene regulatory network in vertebrate development, and a possible correlation between the expression of *lin-28* genes and the *miR-430* family, which is a key regulator of maternal mRNA clearance.

## Discussion

In *C. elegans*, heterochronic genes play a critical role in the timing of organ formation during development [2]–[5]. In this report, we identified zebrafish homologs of *C. elegans lin-28* heterochronic genes and showed that the *C. elegans* heterochronic gene pathway is conserved and could be involved in regulating key temporally controlled events during early zebrafish development. Similar to mammals, zebrafish has two *Lin28* homologs, *lin-28a* and *lin-28b*, in distinct genomic regions. An alignment of the sequences of the zebrafish *lin-28a* and *lin-28b* gene products showed that they are highly homologous with mammalian Lin28a and Lin28b. In particular, two functional domains for *pri-* and *pre-let-7* miRNA binding, a cold-shock domain and a pair of CCHC zinc finger domains, were highly conserved. This high degree of sequence conservation suggests that the function of zebrafish Lin-28a and Lin-28b is conserved across animal phylogeny. Indeed, we observed significant upregulation of *let-7* family miRNA expression in both *lin-28a* and *lin-28b* morphants, implying their conserved function as a *let-7* regulator.

**Figure 6:**
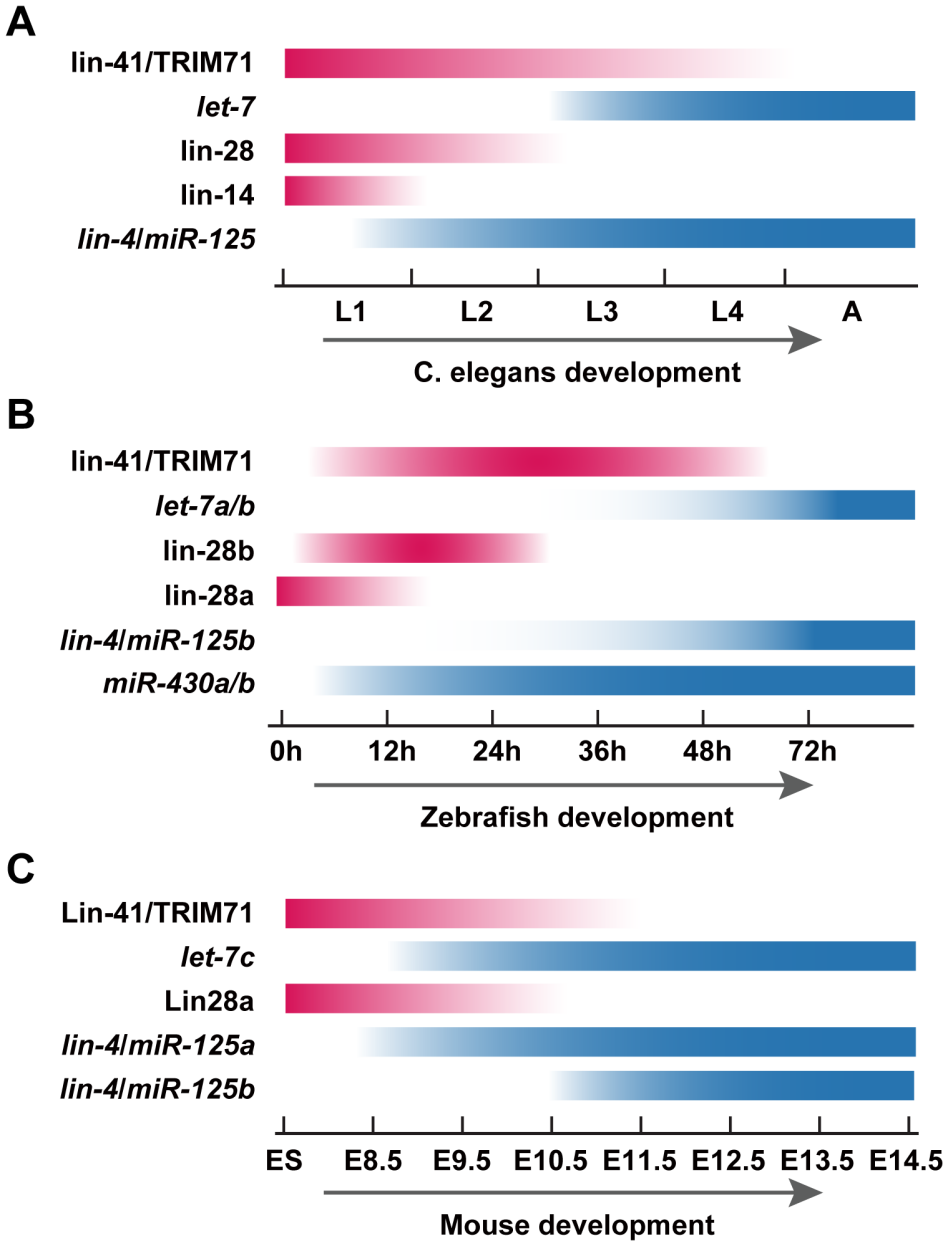
Expression pattern of heterochronic genes in developmental timing. A. Schematic diagram for heterochronic gene expression during *C. elegans* development. Consistent with critical role in the early development, expressions of LIN-14, LIN-28 and LIN-41 were observed at L1 stage. In a reciprocal fashion, Lin-4 and *let-7* expressions were first detected at L1 and L3 stage, respectively and became stronger as development proceeded, thereby down-regulated the expression of their target gene. B. A schematic diagram summarizing heterochronic gene and *miR-430* expression during zebrafish development. As with the expression pattern of *C. elegans* heterechoronic gene, expressions of *lin-28a*, *lin-28b* and *lin-41* were first observed at early development. Reciprocal expression of lin-4 and *let-7* expression were detected at 24 hpf and 48 hpf, respectively, and the expression level increased as development proceeds. Moreover, consistent with the timing of zygotic transcription, *miR-430* expression was activated, and its levels rose. C. Model for heterochronic gene expression during mouse development. The expression of LIN28 was highly restricted in ES cells, which negatively regulated *let-7* expression, and the *let-7* target gene, mouse lin-41, was reported to play an important role as a stem cell specific E3 ubiquitin ligase for the miRNA pathway protein Ago2. As development proceeded, *let-7* and *miR-125a/b* expression levels were increased, and expression levels of Lin28 and Lin41 were prominently downregulated in a reciprocal pattern. Red boxes represent the approximate timing of gene expressions and the blue boxes depicts the timing of the miRNA expressions. Developmental stages of each species are listed at bottom.

During *C. elegans* development, the expression pattern of heterochronic genes is temporally controlled to specify the timing of developmental events [2]–[6]. First, *lin-28* and *lin-41* are expressed at L1. Next, *lin-4* and *let-7* miRNA expression begins at L2 and L3, respectively [5], [6], [13], [14]. Since these miRNAs target the 3′UTR of *lin-28* and *lin-41*, respectively, expression of these genes begins to decrease at L2 and L3, respectively [6], [17], [19] (Figure 6A). Consistent with the sequential and reciprocal expression of heterochronic genes during *C. elegans* larval development, we showed through whole-mount *in situ* hybridization, qRT-PCR and RT-PCR, that the heterochronic gene pathway is highly conserved in zebrafish.

During zebrafish development, although the expression onset was somewhat different, *lin-28a*, *lin-28b*, and *lin-41* were ubiquitously expressed at the early gastrulation stage. Concomitant with the increased expression of *let-7* and *lin-4/miR-125b*, the expression of *lin-28b* and *lin-41* began to decrease from 24 and 48 hpf, respectively (Figure 6B). Since zebrafish *lin-28b* and *lin-41* have a *lin-4/miR-125b* and *let-7* target sites in their 3′UTRs (using Targetscan and PITA) [33], the downregulation of *lin-28b* and *lin-41* might be directly regulated by these miRNAs.

Recent studies have shown that several members of the heterochronic pathway are highly conserved in mammals, not only in sequence but also through regulation by an evolutionarily conserved genetic pathway. For example, *let-7* and *lin-4/miR-125* are highly conserved and expressed in various species, including *Drosophila* and humans [20], [34], [35]. Moreover, *lin-41* and *lin-28* are highly conserved in mammals and are regulated by *let-7* and *miR-125* like *C. elegans* [22], [36].

In mice, Lin28a is a marker of undifferentiated ES cells [24], and is abundant in a variety of developing tissues, including the neuroepithelium at E8.5, and is then expressed throughout the neural tube, co-localized with Sox2 at E9.5 [37]. By E10.5, this expression is markedly decreased in differentiated neural lineages and becomes undetectable [37] (Figure 6C). The mouse Lin41 expression pattern is somewhat similar to that of Lin28a. Lin41/TRIM71 is highly expressed in ES cells [38], and robust and ubiquitous Lin41 expression at E8.5 has been reported [36], [39]. The expression then gradually decreases until E11.0, becoming undetectable at later developmental stages [36], [39] (Figure 6C). On the other hand, *let-7* and *miR-125* are reportedly expressed in a reciprocal fashion to Lin28a and Lin41 during mouse development [36], [39] (Figure 6C).

Considering our and other observations, it seems that an evolutionarily conserved heterochronic gene cascade exists in early vertebrate embryogenesis.

In addition, mammalian homologs of heterochronic genes play a critical roles in the regulation of various undifferentiated cells. For example, in has been suggested that Lin28, *let-7* and *miR125* play important roles in cell fate determination in ES cells [24]. Among others, mammalian homologs of Lin-28 significantly contribute to cancer progression, the pluripotency of embryonic stem (ES) cells, early zygote development and the reprogramming of human and mouse fibroblasts to induced pluripotent stem (iPS) cells [23]–[26], mostly by preventing the anti-proliferative function of *let-7*; thus, the role of Lin-28 in vertebrate development has attracted considerable interest.

Although it has recently been suggested that the Lin28-mediated repression of *let-7*-induced differentiation may play a major role in the maintenance of most undifferentiated cells, increasing evidence strongly suggests the existence of a *let-7*-independent function for Lin28. For example, in neural stem cells, Lin28 regulates the timing of cell fate competency in neural stem cells during neurogliogenesis by a *let-7*-independent mechanism [37].

Consistent with the essential role of *Lin28a* in stem cell maintenance in various types of mammalian cells, we observed a significant decrease in cell proliferation at 5 hpf in both *lin-28a* and *lin-28b* morphant embryos, although a stage-specific cell lineage function of Lin28 remainss to be determined. During the late stage, these morphants displayed a severe phenotype characterized by a shortened body axis, small anterior structures, and aberrant tail morphology. However, while the expression of *let-7a* and *let-7b* was significantly upregulated in these morphants at 24 hpf, we did not find any significant upregulation of these *let-7* miRNAs in these morphants at the early blastula stage (Figure 5B). Thus, it is possible that the *let-7* independent function of Lin-28 might play a critical role in these *lin-28a* and *lin-28b* morphant phenotypes. These findings are consistent with a recent publication in which gross gastrulation defects were observed in *lin-28a* and *lin-28b* morphant *Xenopus* embryos [40]. They did not find any significant changes in the overall level of *let-7* in *lin-28* morphant embryos at the early gastrula stage (10.5), proposing a *let-7*-independent function for Lin-28 (e.g., translational regulation of maternally-deposited mRNAs). Unlike in zebrafish, *lin-28b* is maternally expressed in *Xenopus* and then expression of both *lin-28a* and *lin-28b* is upregulated shortly after mid-blastula transition. Given the fact that the *lin-28a* and *lin-28b* morphants displays similar severe developmental defects in both *Xenopus* and zebrafish, both of the genes seem to be necessary for the early development and not redundant.

Although we were not able to discriminate between maternal *lin-28a* and *lin-28b* function, recent study suggests an intriguing role of Lin28a in mouse early zygote development. Consistent with our results, Vogt et al. reported that *Lin28a* mRNA is maternally expressed, and the Lin28a protein accumulates at the nucleolar precursor body (NPB) of mouse ES cells, where it co-localize with the nucleophosmin1 (NPM1) [26]. MO-mediated knockdown of maternal *Lin28a* inhibit accumulation of NPM1 at presumptive NPB, resulting in a developmental arrest at the transition of the 2-cell to the 4-cell stage and never develop to morula or blastocyst, suggesting that Lin28a is a novel essential factor of nucleologenesis during early zygote development [26]. Further studies are needed to elucidate the functional difference of *lin-28a* and *lin-28b*.

Surprisingly, our microarray-based analysis of miRNA expression in *lin-28a* and *lin-28b* morphants revealed that the expression of *miR-430* family miRNAs was significantly downregulated in these morphants. In the early development of zebrafish, *miR-430* accumulates during the maternal-to-zygotic transition and promotes deadenylation and the clearance of hundreds of maternal mRNAs [12]. Since *miR-430* can rescue the severe developmental defects in MZ *dicer* mutant embryos, *miR-430* is suggested to play a central role in early development [12]. Intriguingly, our *lin-28a* and *lin-28b* morphants displayed a phenotype similar to that of MZ *dicer* mutants, which is characterized by reduced extension of the body axis, resulting in shortening of the embryo and morphological malformation of the heart and brain. Thus, we speculate that the function of Lin-28 during early zebrafish development involves the *miR-430*-mediated clearance of maternal mRNAs. Currently, we do not have an explanation for the genetic interaction between *lin-28* and *miR-430*. It would be important to consider this interaction in future experiments.

We also observed the expression of *lin-41,* a down-stream target of *let-7* in the presumptive anterior neural plate. Since it was downregulated in *lin-28a* and *lin-28b* morphant embryos, there may exist a conserved functional role of heterochronic gene cascade in zebrafish. In mouse ES cells, Lin41 regulates *let-7* activity in cooperation with Lin28 and acts as a stem cell-specific E3 ubiquitin ligase [38]. Moreover, Lin28 is expressed in developing mouse neural tube, co-localizes with SOX2, and has been demonstrated to play a role in neurogenesis [37]. Lin41-knockout mice display a striking neural tube closure defect during development and embryonic lethality [39]. Thus, these results suggest that a significant gene regulatory network exists between *Lin28*, *Sox2*, and *Lin41* during early vertebrate embryogenesis.

Based on homology and expression pattern, zebrafish *lin-28a*, *lin-28b*, and the miRNAs studied here may be involved in regulating specific genes directing key developmental events. These findings will aid in the understanding of the evolutionarily conserved roles of the heterochronic gene cascade underlying early embryonic development in vertebrates.

## Supporting Information

Figure S1 **Genomic organization of *LIN28A/B* and its homologous locus in various species.** VISTA plot of the human *LIN28A* (A) and *LIN28B* (B) genomic sequence (x-axis) vs. mouse, rat, chicken and zebrafish genomic sequence (y-axis). Regions of high conservation are colored according to the annotation as exons (dark blue), untranslated regions (light blue) or non-coding (pink).
(EPS)
